# Author Correction: Ex vivo anticoagulants affect human blood platelet biomechanics with implications for high-throughput functional mechanophenotyping

**DOI:** 10.1038/s42003-022-03130-4

**Published:** 2022-02-23

**Authors:** Laura Sachs, Jan Wesche, Lea Lenkeit, Andreas Greinacher, Markus Bender, Oliver Otto, Raghavendra Palankar

**Affiliations:** 1 Institute for Immunology and Transfusion Medicine, University Medicine Greifswald, Fleischmannstr.8, 17475 Greifswald, Germany; 2 Institute of Experimental Biomedicine - Chair I, University Hospital and Rudolf Virchow Center, Würzburg, Germany; 3 Zentrum für Innovationskompetenz – Humorale Immunreaktionen bei Kardiovaskulären Erkrankungen, Universität Greifswald, Fleischmannstr. 42, 17489 Greifswald, Germany; 4 Deutsches Zentrum für Herz-Kreislauf-Forschung e.V., Standort Greifswald, Universitätsmedizin Greifswald, Fleischmannstr. 42, 17489 Greifswald, Germany

**Keywords:** Myosin, Lab-on-a-chip, High-throughput screening

Correction to: *Communications Biology* 10.1038/s42003-021-02982-6, published online 21 January 2022.

The original version of this Article contained an error in Fig. 4d, in which a representative microscopy image for TRAP-6 stimulated platelets in Li-Heparin was erroneously used for the K2-EDTA panel. The correct version of Fig. 4 is:
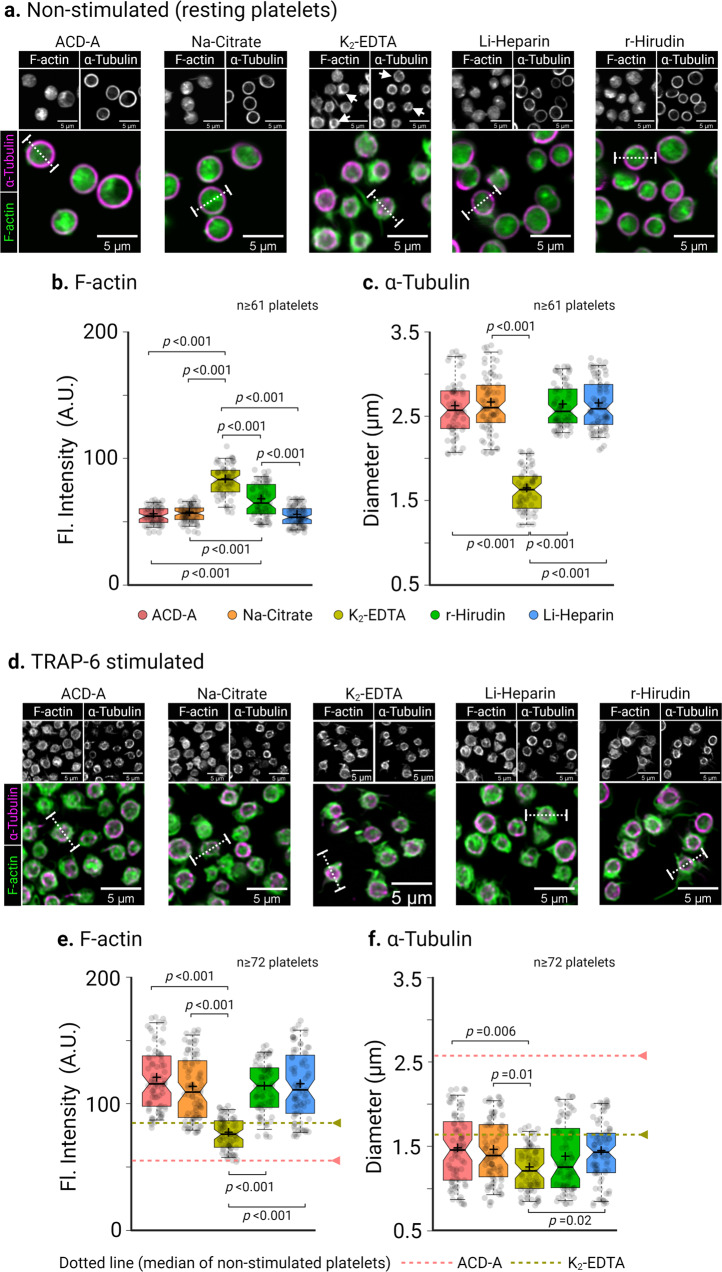


which replaces the previous incorrect version.
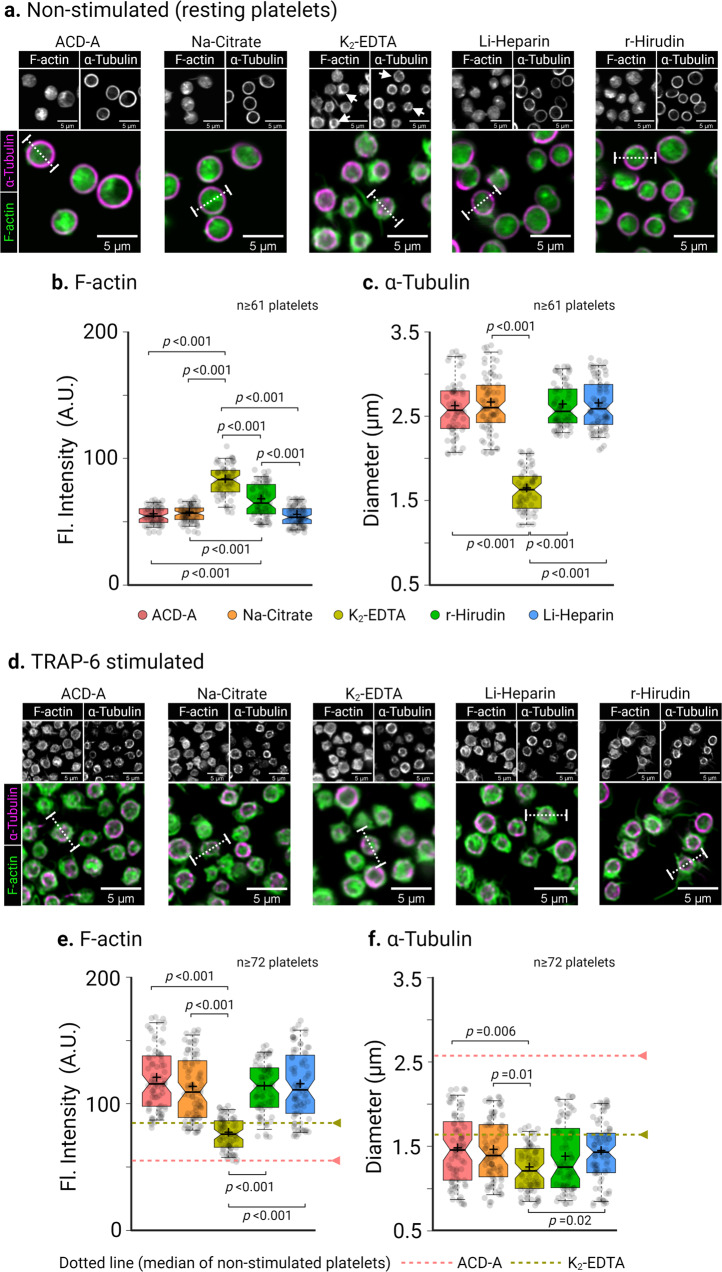


The link to the raw data has been updated in the Data Availability statement to: ‘10.5281/zenodo.4461273.

The error has been corrected in both the PDF and HTML versions of the Article.

