# Supplementary material for: Ex vivo anticoagulants affect human blood platelet biomechanics with implications for high-throughput functional mechanophenotyping
Source: Commun Biol. 2022 Jan 21;5:86. doi: 10.1038/s42003-021-02982-6 (PMC8782918; doi:10.1038/s42003-021-02982-6)
Supplement: Supplementary file 3 — Description of Additional Supplementary Files [file 42003_2021_2982_MOESM3_ESM.pdf]

Description of Additional Supplementary Files

File name: Supplementary Data 1

Description: Captions for source data in Excel format corresponding to individual figures in the main text are as follows,

Figure 1-Source Data Platelet deformation and size.

Figure 2-Source Data Platelet deformation and CD62P surface expression.

Figure 3-Source Data Platelet deformation and PAC-1 binding.

Figure 4-Source Data Platelet F-actin and tubulin distribution.

Figure 5-Source Data Changes in F-actin content.

Figure 6-Source Data Impact of LatB on platelet deformation.

Figure 7-Source Platelet mechanophenotype in MYH9 patient.
