# Supplementary material for: Ex vivo anticoagulants affect human blood platelet biomechanics with implications for high-throughput functional mechanophenotyping
Source: Commun Biol. 2022 Jan 21;5:86. doi: 10.1038/s42003-021-02982-6 (PMC8782918; doi:10.1038/s42003-021-02982-6)
Supplement: Supplementary file 5 — Reporting Summary [file 42003_2021_2982_MOESM5_ESM.pdf]

nature portfolio

Corresponding author(s): Raghavendra Palankar

Last updated by author(s): Nov 23, 2021

Reporting Summary

Nature Portfolio wishes to improve the reproducibility of the work that we publish. This form provides structure for consistency and transparency in reporting. For further information on Nature Portfolio policies, see our Editorial Policies and the Editorial Policy Checklist.

Statistics

For all statistical analyses, confirm that the following items are present in the figure legend, table legend, main text, or Methods section.

n/a	Confirmed	
[ ]	[x]	The exact sample size (*n*) for each experimental group/condition, given as a discrete number and unit of measurement
[ ]	[x]	A statement on whether measurements were taken from distinct samples or whether the same sample was measured repeatedly
[ ]	[x]	The statistical test(s) used AND whether they are one- or two-sided<br>*Only common tests should be described solely by name; describe more complex techniques in the Methods section.*
[x]	[ ]	A description of all covariates tested
[x]	[ ]	A description of any assumptions or corrections, such as tests of normality and adjustment for multiple comparisons
[ ]	[x]	A full description of the statistical parameters including central tendency (e.g. means) or other basic estimates (e.g. regression coefficient) AND variation (e.g. standard deviation) or associated estimates of uncertainty (e.g. confidence intervals)
[ ]	[x]	For null hypothesis testing, the test statistic (e.g. *F*, *t*, *r*) with confidence intervals, effect sizes, degrees of freedom and *P* value noted<br>*Give P values as exact values whenever suitable.*
[x]	[ ]	For Bayesian analysis, information on the choice of priors and Markov chain Monte Carlo settings
[x]	[ ]	For hierarchical and complex designs, identification of the appropriate level for tests and full reporting of outcomes
[x]	[ ]	Estimates of effect sizes (e.g. Cohen's *d*, Pearson's *r*), indicating how they were calculated

Our web collection on statistics for biologists contains articles on many of the points above.

Software and code

Policy information about availability of computer code

Data collection	RT-FDC data was acquired using the ShapeIn software (Version 2.0, Zellmechanik Dresden, Germany)<br>Flow cytometry data was collected using CXP Cytometry List Mode Dada Acquisition & Analysis Software(Version 2.3, Beckmann Coulter).<br>Confocal Laser Scanning Microscopy data was acquired on Leica Application Suite X (LAS X).
Data analysis	RT-FDC data were exported as fcs files after entering the range area ratio (0-1.1) and fluorescence maximum crosstalk compensation in the Shape-Out analysis software (https://github.com/ZELLMECHANIK-DRESDEN/ShapeOut2/releases/tag/2.3.0 Version 2.3, Zellmechanik Dresden, Germany). The data were analyzed using FlowJo™ software for Windows, Version v10.6.2. (Becton, Dickinson and Company, USA) with platelet size gate (0-10 µm). Gating s<br>Flow cytometry results were analyzed using FlowJo™ software for Windows, Version v10.6.2. (Becton, Dickinson and Company, USA) with CD61 Gating for platelet indicator.<br>Image analysis was performed on ImageJ 2.1.0/1.53c; Java 1.8.0_172 [64-bit]; for Windows 10.<br>For all data plotting and statistical analyses, GraphPad Prism version 8.0.0 for Windows (GraphPad Software, San Diego, California USA) was used.

For manuscripts utilizing custom algorithms or software that are central to the research but not yet described in published literature, software must be made available to editors and reviewers. We strongly encourage code deposition in a community repository (e.g. GitHub). See the Nature Portfolio guidelines for submitting code & software for further information.

Data

Policy information about availability of data

All manuscripts must include a data availability statement. This statement should provide the following information, where applicable:

- Accession codes, unique identifiers, or web links for publicly available datasets
- A description of any restrictions on data availability
- For clinical datasets or third party data, please ensure that the statement adheres to our policy

All datasets are available at publicly accessible via Zenodo at the following DOI: 10.5281/zenodo.4461272 (https://zenodo.org/record/4461273#.YJOcV7UzYUE)

Field-specific reporting

Please select the one below that is the best fit for your research. If you are not sure, read the appropriate sections before making your selection.

[x] Life sciences [ ] Behavioural & social sciences [ ] Ecological, evolutionary & environmental sciences

For a reference copy of the document with all sections, see nature.com/documents/nr-reporting-summary-flat.pdf

Life sciences study design

All studies must disclose on these points even when the disclosure is negative.

Sample size	The research has a pool of healthy donors. Whole blood from 6 healthy donors was tested on different days. The whole blood of the MYH9 patient and from the healthy donor was collected on one day .
Data exclusions	No data were excluded from the analysis.
Replication	The experiment was repeated six times with healthy donors.
Randomization	not applicable
Blinding	not applicable

Reporting for specific materials, systems and methods

We require information from authors about some types of materials, experimental systems and methods used in many studies. Here, indicate whether each material, system or method listed is relevant to your study. If you are not sure if a list item applies to your research, read the appropriate section before selecting a response.

Materials & experimental systems

n/a	Involved in the study	
[ ]	[x]	Antibodies
[x]	[ ]	Eukaryotic cell lines
[x]	[ ]	Palaeontology and archaeology
[x]	[ ]	Animals and other organisms
[ ]	[x]	Human research participants
[x]	[ ]	Clinical data
[x]	[ ]	Dual use research of concern

Methods

n/a	Involved in the study	
[x]	[ ]	ChIP-seq
[ ]	[x]	Flow cytometry
[x]	[ ]	MRI-based neuroimaging

Antibodies

Antibodies used	PE conjugated CD61 (Clone SZ21, Cat. No. IM3605, Beckman Coulter), AlexaFluor647 conjugated CD62P (Clone AK4, Cat.No. 304918, BioLegend, USA), FITC conjugated PAC1 (Clone PAC-1, Cat. No. 340507, B.D. Biosciences, USA), FITC conjugated CD62P (Cat. No. A07790, Beckman Coulter), Phalloidin-Atto-647N (Cat. No. AD647N-81, Atto-Tec GmbH, Germany), mouse monoclonal anti-α-Tubulin IgG (Clone DM1A, Cat.No. T9026, Sigma Aldrich GmbH, Germany), Donkey anti-mouse IgG H&L-AlexaFluor488 (Cat. No. ab150109, abcam)
Validation	Commercially available antibodies that are standard for platelet function studies

Human research participants

Policy information about studies involving human research participants

Population characteristics	The donors had not taken any medication in the previous ten days before blood collection.
Recruitment	Healthy donors are part of the research donor pool.
Ethics oversight	The use of platelet-rich plasma (PRP) from healthy adult individuals and MYH9 patients was approved by the ethics committee of the University Medicine Greifswald, Germany. All participants gave written, informed consent.

Note that full information on the approval of the study protocol must also be provided in the manuscript.

Flow Cytometry

Plots

Confirm that:

- [x] The axis labels state the marker and fluorochrome used (e.g. CD4-FITC).
- [x] The axis scales are clearly visible. Include numbers along axes only for bottom left plot of group (a 'group' is an analysis of identical markers).
- [x] All plots are contour plots with outliers or pseudocolor plots.
- [x] A numerical value for number of cells or percentage (with statistics) is provided.

Methodology

Sample preparation	Whole blood was collected by venipuncture in BD Vacutainer® Tubes containing acid citrate dextrose solution A (ACD-A), 3.8% buffered trisodium citrate (Na-Citrate), 102 I.U. Lithium-Heparin (Li-Heparin), 1.8mg/mL dipotassium ethylenediaminetetraacetic acid (K2 -EDTA), or 171 ATU/mL recombinant hirudin (r-Hirudin) (REVASC, Canyon Pharmaceuticals, USA). Whole blood was stored at room temperature for 15 min (at 45° angle to the horizontal surface) and then centrifuged (120 x g for 20 min at room temperature). Platelet rich plasma (PRP) was transferred to a new polypropylene tube and incubated for 15 min at 37°C.<br><br>20 µl stained PRP (CD61-PE (Cat. No. IM3605, Beckman Coulter), CD62P-FITC (Cat. No. A07790, Beckman Coulter)) are incubated with 2.2 µl PBS (Cat.No. P04-36500, PAN Biotech GmbH, Germany) or TRAP-6 (Haemochrom Diagnostica GmbH, Germany) for 10 min (RT protected from light). PRP is fixed with 2.5 µl buffer-1 (Cat. No. B31168, PerFix-NC, Beckman Coulter) and incubated for 15 min (RT protected from light). 150 µl buffer 2 from the PerFix kit (containing Phalloidin-Atto-647N (Cat. No. AD647N-81, Atto-Tec GmbH, Germany)) is added and incubated for 30 min (RT protected from light). 1.5 ml of buffer-3 from the PerFix Kit is added.
Instrument	Cytomics FC 500
Software	Flow cytometry data was collected using CXP Cytometry List Mode Dada Acquisition & Analysis Software(Version 2.3, Beckmann Coulter). Flow cytometry results were analyzed using FlowJo™ software for Windows, Version v10.6.2. (Becton, Dickinson and Company, USA)
Cell population abundance	not applicable
Gating strategy	Human platelet CD61 as a platelet specific marker in RT-FDC and FACS

- [x] Tick this box to confirm that a figure exemplifying the gating strategy is provided in the Supplementary Information.
